# Impact of rifampicin on P-glycoprotein (*ABCB1*) expression in M1 and M2 macrophages derived from the THP-1 monocytic cell line or peripheral blood mononuclear cells

**DOI:** 10.1007/s00210-026-05231-x

**Published:** 2026-03-25

**Authors:** Katharina Hamburg, Anna Staszelis, Camilo Scherkl, Johanna Weiss, Julia Carolin Stingl, Dirk Theile

**Affiliations:** Internal Medicine IX - Dept. of Clinical Pharmacology & Pharmacoepidemiology, Heidelberg University, Medical Faculty of Heidelberg, Im Neuenheimer Feld 410, 69120 Heidelberg, Germany

**Keywords:** P-glycoprotein, Peripheral blood mononuclear cells, Rifampicin, *Mycobacterium tuberculosis*

## Abstract

Rifampicin induces ABCB1 (coding for P-glycoprotein; P-gp) in enterocytes or hepatocytes, lowering drug levels and efficacy. However, there is no induction data for macrophages, the primary site of rifampicin’s anti-tuberculosis action. THP-1 cells were differentiated (200 nM phorbol 12-myristate-13-acetate; 72 h) and polarized to M1 macrophages (50 ng/mL lipopolysaccharide (LPS), 20 ng/mL interferon-gamma (IFNγ); 48 h) or M2 macrophages (20 ng/mL interleukin 4 (IL-4) and interleukin 13 (IL-13) each; 48 h). Peripheral blood mononuclear cells (PBMC) from a healthy volunteer were differentiated using a commercial kit and polarized to M1 (10 ng/mL LPS and 50 ng/mL IFNγ; 72 h) or to M2 cells (20 ng/mL IL-4 and IL-13 each; 72 h). Polarized macrophages were exposed to 10 µM rifampicin for 1 week and mRNA levels of ABCB1, ABCG2 (coding for breast cancer resistance protein), and SLCO2B1 (coding for organic anion-transporting polypeptide 2B1) were evaluated by quantitative real-time polymerase chain reaction. P-gp protein levels were evaluated by Western blot analysis. Rifampicin enhanced ABCB1 in THP-1-derived M1 cells (fivefold) and M2 cells (sixfold; protein increased by 50%), but not in PBMC-derived macrophages (linear regression model). However, this effect was not significant in the three-way ANOVA, given the strong influence of macrophage polarization (M1 vs. M2) and macrophage source (THP-1 vs. PBMC). ABCG2 was enhanced twofold in THP-1-derived M2 cells (linear regression model), while SLCO2B1 remained unaffected by rifampicin. In conclusion, ABCB1 expression in macrophages differs by the cell model (THP-1 cell line vs. primary PBMC) and the polarization phenotype (M1 vs. M2). Strong rifampicin-mediated enhancements of ABCB1 were only observed in THP-1-derived M1 and M2 cells.

**Supplementary Information:**

The online version contains supplementary material available at 10.1007/s00210-026-05231-x.

## Introduction

Rifampicin is an important antibiotic, mostly used to treat infections with *Mycobacterium tuberculosis* and the related tuberculosis disease. While being highly efficient, the pharmacotherapy with rifampicin is characterized by its high drug interaction potential with the risk to dramatically lower the plasma levels of co-administered drugs. For instance, the plasma concentrations of itraconazole (Jaruratanasirikul et al., 1998), voriconazole (Geist et al. 2007), indinavir (Avihingsanon et al. 2012), or telaprevir (Garg et al. 2013) are lowered by more than 90% after the repetitive administration of 600 mg rifampicin. This results from rifampicin-mediated transcriptional induction of drug-metabolizing enzymes and drug transporters in enterocytes and hepatocytes, eventually altering drug absorption and elimination of the co-administered compounds mentioned (Paine et al. 2006; Gundert-Remy et al. 2014; Lin 2003). In detail, P-glycoprotein (P-gp, encoded by *ABCB1*) is an ATP-driven transmembrane drug transporter that is located at these pharmacologically important barriers, modulating intestinal uptake and distribution of drugs and mediating drug-drug interactions (Lin 2003).

Besides its relevance for systemic drug concentrations, P-gp also limits the uptake of its substrates into immune cells (e.g., monocytes, macrophages), which are the anti-tuberculosis drug treatment sites of action (Gupta & Gollapudi 1993). Accordingly, rifampicin and combinational anti-tuberculosis drugs’ efficacy may be affected if rifampicin exhibited the same inducing effects in immune cells as observed for enterocytes and hepatocytes. However, there is contradicting or incomplete data on this issue. The contradiction relates to the many different methodological approaches that were used to evaluate rifampicin’s effects. On the one hand, in vitro investigations in monocyte-representing cell lines failed to demonstrate any inducing effects (Manceau et al. 2010). Alternatively, ex vivo experiments exposed peripheral blood mononuclear cells (PBMC) to rifampicin and showed moderate *ABCB1*/P-gp enhancements (Manceau et al. 2010; Owen et al. 2006). On the other hand, in vivo investigations treated healthy volunteers with rifampicin, obtained their PBMC post-treatment, and recorded some iatrogenic changes of *ABCB1* expression in PBMC (Asghar et al. 2002). Together, the observed contradiction likely originates from the diverse routes of rifampicin exposure of monocytes *(*in vitro, ex vivo, in vivo). The gross data incompleteness, in turn, refers to the non-existing data in immune cells other than monocytes, e.g., macrophages. This is a relevant scientific gap because macrophages are the main hosts for *Mycobacterium tuberculosis* (Bold & Ernst 2009) and thus the most important target cells for rifampicin and other anti-tuberculosis antibiotics being transported by P-gp such as bedaquiline (Kotwal et al. 2020) or moxifloxacin (Brillault et al. 2009). Given this uncertainty, this study for the first time aimed at systematically evaluating the drug transporter-modulating effects of rifampicin treatment (1 week exposure to 10 µM) on both pro-inflammatory M1 macrophages and anti-inflammatory M2 macrophages, respectively derived from a well-known cell line (THP-1 monocytes) or PBMC (from a healthy volunteer).

## Materials and methods

### Materials

THP-1 cells were obtained from the American Type Culture Collection (ATCC, Manassas, USA). The L-MDR1 cell line (overexpressing human *ABCB1*/P-gp) had been provided by A.H. Schinkel (The Netherlands Cancer Institute, Division of Experimental Therapy, Amsterdam, The Netherlands) (Schinkel et al. 1996). RPMI 1640 medium and medium 199 were purchased from PanBiotech (Aidenbach, Germany). Media for monocyte isolation from PBMCs, M1 and M2 macrophage differentiation were purchased from PromoCell™. Dulbecco’s phosphate buffered saline (DPBS), penicillin–streptomycin, fetal calf serum (FCS), phorbol-12-myristate-13-acetate (PMA), interferon gamma (IFNγ), lipopolysaccharides (LPS) from *E. coli,* interleukin 4 and 13, and rifampicin were purchased from Sigma-Aldrich (Taufkirchen, Germany). The RevertAiD h Minus First Strand cDNA Synthesis Kit, the RIPA buffer, the Pierce™ BCA™ Protein-Assay-Kit, the Pierce™ ECL Western Blotting-Substrate, the anti-P-gp antibody (Clone C219, Invitrogen #MA1-26528), and the ECL™ anti-mouse secondary IgG antibody (NXA931V) were bought from Thermo Fisher (Dreieich, Germany). The β-Actin antibody (C4, sc-47778) was purchased from Santa Cruz Biotechnology (Dallas, USA). Protease inhibitors pefabloc, leupeptin, pepstatin and aprotinin were purchased from Carl Roth (Karlsruhe, Germany). ABSOLUTE™ QPCR SYBR® Green Mix was from ABgene (Epsom, United Kingdom). 4 × Laemmli-Puffer was brought from Bio-Rad (Feldkirchen, Germany). Dithiothreitol (DDT) was purchased from AppliChem (Darmstadt, Germany). Cytiva Ficoll-Paque™ PLUS and Greiner Bio-One Leucosep™ tubes were purchased from Thermo Fisher Scientific (Schwerte, Germany).

### Isolation of monocytes from peripheral blood mononuclear cells and their differentiation and polarization

After written informed consent, whole blood was drawn from a healthy male volunteer, who was free of any medication. The study was approved by the responsible Ethics Committee of the Medical Faculty of Heidelberg University (S-384/2016). Blood samples (8 × 8 mL tubes) were transferred into four Leucosep™ tubes and centrifuged for 15 min at 800 × g and room temperature (RT) with no brake (prevention of stirring-up separated PBMCs). The supernatant was removed and the pellet containing PBMCs was washed two times with DPBS and pelleted again by centrifugation at 300 × g for 8 min (RT; with brake). The obtained pellet was resuspended again in DPBS and the cell count of monocytes contained therein was determined using a flow cytometer (MACSQuant analyzer 10, Miltenyi Biotec, Bergisch Gladbach, Germany). Isolation of monocytes from PBMCs was performed as described in the manufacturer’s protocol. PBMCs (1 × 10^6^ cells per flask) were put in cell culture flasks using monocyte attachment media. After 1 h of incubation, media was removed and cells were washed with the same media, removing floating cells and enriching attached monocytes. Those were either treated with M1- or M2-generation media and incubated for 6 days at 5% CO_2_ and 37 °C. After this period, obtained macrophages were polarized for 72 h (5% CO_2_ and 37 °C) using either 10 ng/mL LPS and 50 ng/mL IFNγ (leading to M1 phenotype) or 20 ng/mL IL-4 and IL-13 each (M2 phenotype).

### THP-1 cells and their differentiation and polarization

THP-1 cells were cultured in RPMI 1640 medium supplemented with 10% FCS and penicillin (100 U/mL)-streptomycin (0.1 mg/mL) at 5% CO_2_ and 37 °C. Differentiation and polarization were performed as described previously (Hamburg et al. 2026). Briefly, THP-1 monocytes were initially differentiated into M0 macrophages using 200 nM PMA for 72 h. After a recovery phase of 5 days, M0 macrophages were polarized for 48 h either into M2 cells by treating them with 20 ng/mL IL-4 and IL-13 each or into M1 cells using 50 ng/mL LPS and 20 ng/mL IFNγ. This protocol has been demonstrated to lead to correctly polarized THP-1-derived macrophages, characterized by the overexpression of specific M1 (e.g., tumor-necrosis factor alpha, TNFα) or M2 markers (e.g., macrophage mannose-receptor C-type 1, MRC-1) (Hamburg et al. 2026).

### Drug exposure

Obtained polarized macrophages (M1, M2) from both sources (THP-1, PBMC) were exposed for 1 week with 10 µM rifampicin, dissolved in the respective culture medium. During this incubation week, cells were constantly kept in the dark at 5% CO_2_ and 37 °C.

### Drug transporter mRNA expression evaluation

Total RNA was isolated from all samples using the GeneElute Mammalian Total RNA Miniprep Kit according to the manufacturer’s instructions. cDNA was synthesized from total RNA by using the RevertAid™ H Minus First Strand cDNA Synthesis Kit. A random hexamer primer was used according to the manufacturer’s instructions. Expression levels of *ABCB1, ABCG2*, and *SLCO2B1* were quantified by real-time reverse transcription (RT) polymerase chain reaction (PCR) with a LightCycler® 480 (Roche Applied Science, Mannheim, Germany) as described previously (Albermann et al. 2005; Weiss et al. 2011). Primer sequences for *ABCB1* (Albermann et al. 2005), *ABCG2* (Sauerbrey et al. 2002), *SLCO2B1* (König et al. 2010), *GU* (glucuronidase-beta; Zisowsky et al. 2007), and *RPL13* (large ribosomal sub-unit 13; Zisowsky et al. 2007) are displayed in Table 1. The housekeeping genes GU and RPL13 were used for normalization (Hamburg et al. 2026). Data was analyzed from three independent biological replicates (e.g., blood drawings) with technical duplicates (PCR runs) each as described previously (Albermann et al. 2005).

**Table 1:** Primer sequences of tested drug transporters and reference genes

Genes	Forward primer	Reverse primer	Reference
*ABCB1*	5′- CCCATCATTGCAATAGCAGG	5′- TGTTCAAACTTCTGCTCCTGA	Albermann et al. (2005)
*ABCG2*	5′- AGATGGGTTTCCAAGCGTTCAT	5′- CCAGTCCCAGTACGACTGTGACA	Sauerbrey et al. (2002)
*SLCO2B1*	5′- CGCACTCACTGATTCCTACACATT	5′- AAGCCATAGCATCACTCATTACAC	König et al. (2010)
*GU*	5´-TTCACCAGGATCCACCTCTG	5´-TGTTCAAACTTCTGCTCCTG	Zisowsky et al. (2007)
*RPL13*	5´-GCTCATGAGGCTACGGAAAC	5´-TATTGGGCTCAGACCAGGAG	Zisowsky et al. (2007)

### P-gp protein expression evaluation

P-gp protein expression in rifampicin-treated and untreated THP-1-derived macrophages or L-MDR1 cells (positive control) was evaluated by Western blotting. L-MDR1 cells, untreated macrophages, and rifampicin-treated macrophages were initially washed with PBS, centrifuged for 5 min at 400 g and lysed with RIPA buffer (containing 1 mg/mL pefabloc, 5 μg/mL leupeptin, 1 μg/mL pepstatin and 1 μg/mL aprotinin). The Pierce BCA™ Protein-Assay-Kit was used to determine protein concentrations in the samples. Proteins were denaturized and diluted in 5 × SDS buffer (4 × LaemmLi-Puffer + 1 × 1 M DTT) to reach concentrations of 10 µg/mL per sample. Samples were run on a SDS–polyacrylamide gel for 120 min (stacking gel: 4%; separation gel: 10%) and subsequently blotted to a nitrocellulose membrane for 70 min and incubated in blocking buffer (5% BSA Tris-buffered saline with 0.1% Tween 20) for 30 min. Membranes were exposed to primary monoclonal antibodies overnight at 4 °C (Tris-buffered saline with 0.1% Tween 20; anti-P-gp antibody diluted 1:100, anti-β-actin antibody diluted 1:2000), followed by incubation with the anti-mouse IgG horseradish peroxidase-conjugated secondary antibody (1:2000) and interim washing steps. Protein bands were stained and exposed to the Pierce™ ECL Western Blotting-Substrate on radiographic film for 1–5 min. Relative P-gp expression was semi-quantitatively evaluated by densitometry (ImageJ, Bethesda, USA). Lanes from top to bottom of each sample were marked and histograms of signal intensities were recorded. Signals from P-gp were normalized to signals from β-actin, and the ratio of rifampicin-exposed samples was normalized to the ratio in unexposed cells (set to 1).

### Statistical analysis

The statistical analysis was performed using the GraphPad Prism 9.00 software (GraphPad Software, San Diego, USA). For statistics regarding exposure-related mRNA expression changes in M1 or M2 cells, either derived from THP-1 cells or PBMC, a three-way ANOVA with Šídák’s test (correcting for multiple comparisons) was used. For the selected evaluation of the treatment effect in the particular macrophage type, a linear regression model depending on rifampicin exposure with the following equation ${Y}_{j}= {\beta }_{0} + {\beta }_{treat}{X}_{treat} +{\epsilon }_{j}$ was used additionally. The linear regression model was conducted with R version 4.3.1 (R Foundation for Statistical Computing, Vienna, Austria). For determination of polarization markers and the P-gp protein levels in THP-1-derived macrophages (rifampicin exposure vs. untreated contols), an unpaired *t* test with Welch´s correction was used. A P value < 0.05 was considered significant.

## Results

### Differentiation and polarization of PBMC-derived M1 and M2 macrophages

**Fig. 1:**
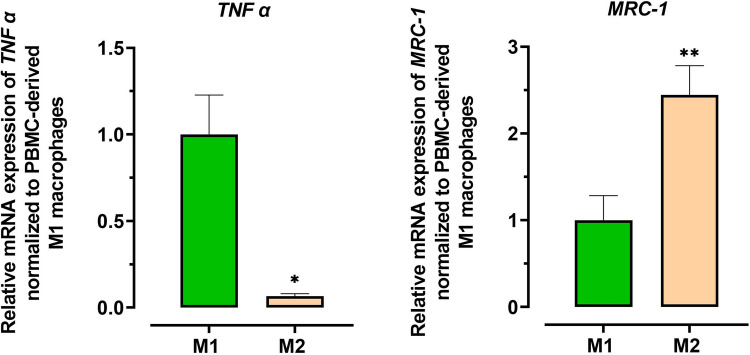
Relative mRNA expression of selected polarization markers in PBMC-derived M1 macrophages (green) or M2 macrophages (light orange). Data was normalized to mRNA expression of M1 macrophages and are shown as mean ± S.D. of three independent experiments. Statistical significance was determined by unpaired t test with Welch’s correction. A *P* value < 0.05 was considered significant. *P < 0.05; **P < 0.01

THP-1 monocyte differentiation and polarization were performed according to our previously published standard protocol and led to correctly polarized macrophages (data already published in Hamburg et al. 2026). In contrast, obtaining M1 cells and M2 cells from primary PBMC is less standardized and reliable. Accordingly, mRNA expression of validated polarization markers in these PBMC-derived M1 and M2 macrophages was analyzed by PCR (Fig. 1). The pro-inflammatory marker TNFα was significantly higher expressed in M1 macrophages (set to 1) than in M2 macrophages (*P* < 0.05). In contrast, the anti-inflammatory marker gene MRC-1 was significantly increased in M2 macrophages (*P* < 0.01) compared to M1, confirming the correct phenotype of the obtained PBMC-derived macrophage populations.

### Impact of cell source, cell type, and rifampicin exposure on drug transporter expression

**Fig. 2:**
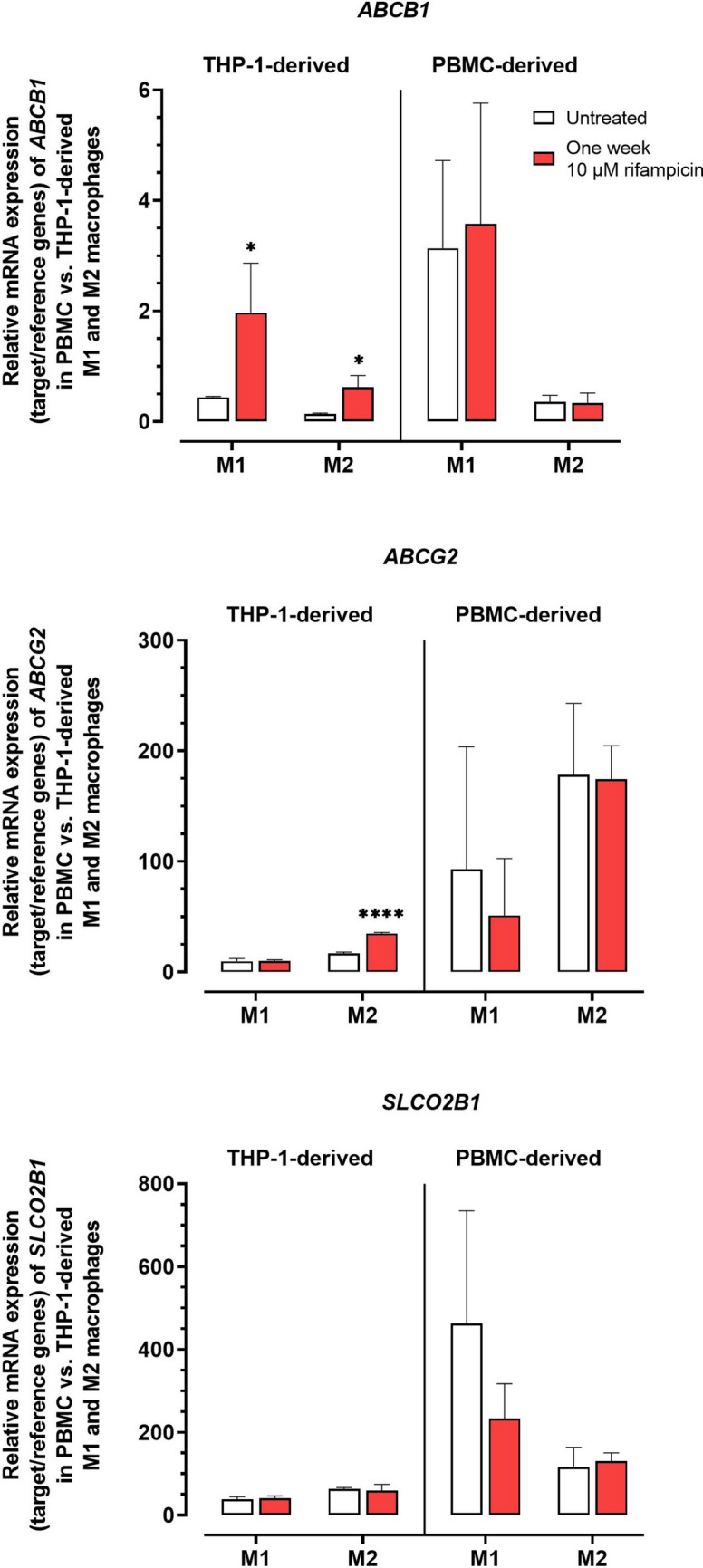
Relative mRNA expression of drug transporters in PBMC- and THP-1-derived M1 and M2 macrophages, either left untreated (white bars) or after 1 week of 10 µM rifampicin exposure (red bars). For statistical analysis, a three-way ANOVA with Šídák’s test (correcting for multiple comparisons) was used. Data are shown as mean ± S.D. of three independent experiments. For the selected evaluation of the treatment effect in the particular macrophage type, a linear regression model was performed, considering a *P* value < 0.05 significant. *P < 0.05; ****P < 0.0001

We compared mRNA expression levels of the drug transporters *ABCB1, ABCG2,* and *SLCO2B1* between M1 and M2 cells (cell type), either derived from PBMC or THP-1 cells (cell source), in both non-exposed and rifampicin-exposed conditions (Fig. 2). A three-way ANOVA was performed to investigate which of these factors (cell type, cell source, or rifampicin treatment) has the most profound influence on drug transporter expression.

**Fig. 3:**
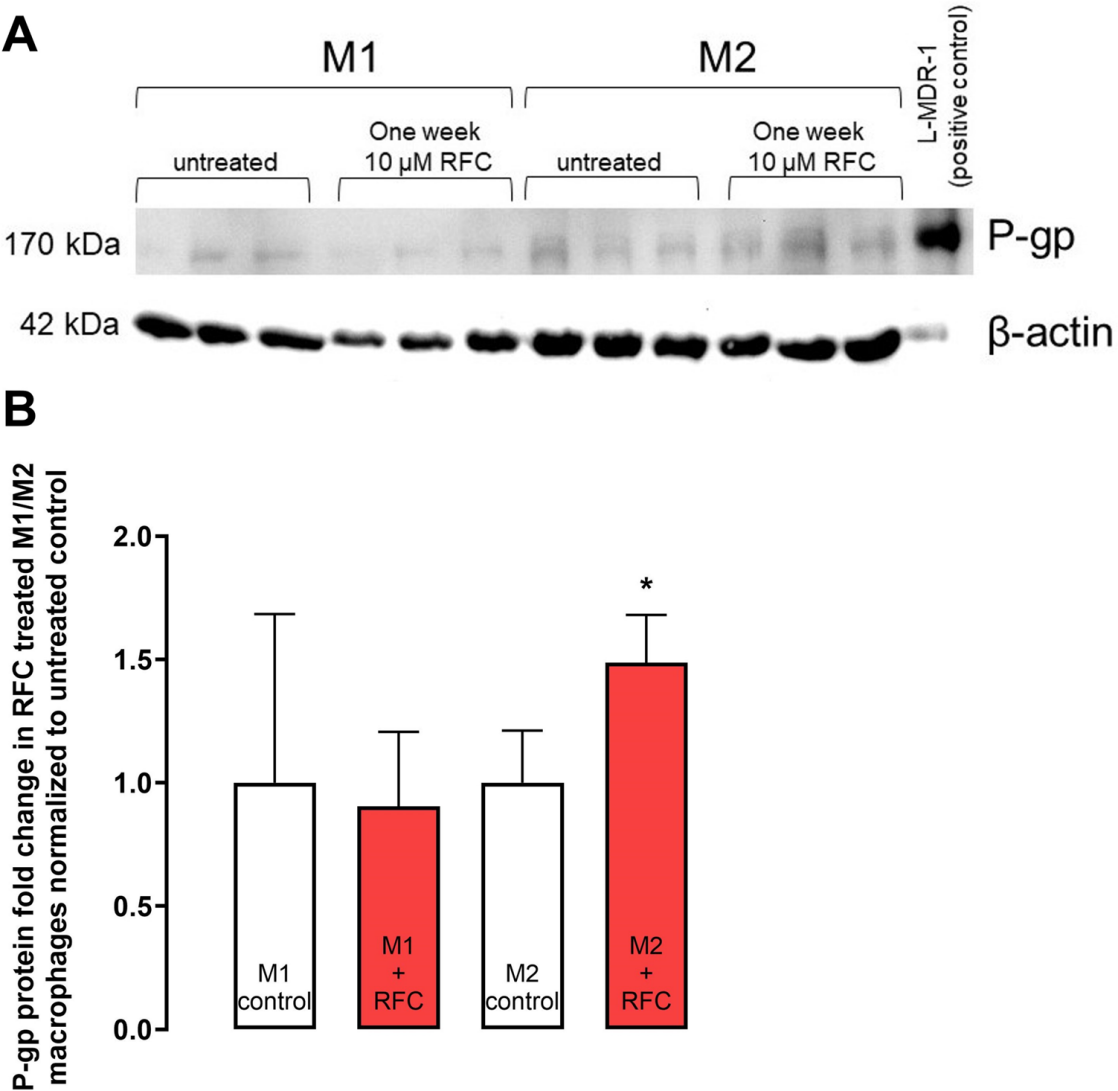
**A** Western blot membrane showing expression of P-gp (170 kDa) and β-actin (42 kDa, loading control) in treated (10 µM rifampicin for 1 week) and untreated M1 macrophages and M2 macrophages, derived from THP-1 monocytes. Genetically engineered L-MDR1 cells with high P-gp overexpression served as a control (far right lane). **B** Semi-quantitative analysis was performed using ImageJ, normalizing signals from P-gp to signals from ß-actin. Resulting ratios of rifampicin (RFC)-treated cells were normalized to untreated controls of the corresponding cell type. Data are shown as the mean ± S.D. of the P-gp/β-actin ratios of the biological triplicates of each condition. Statistical significance was determined by parametric Welch’s test

For *ABCB1,* the cell type strongly affected the mRNA expression level. This means that the most significant difference (*P* < 0.001) was observed between M1 macrophages (high expression) and M2 macrophages (low expression). Additionally, the cell source (PBMC vs. THP-1) was a relevant contributor as well, but with lower significance (*P* < 0.05). Thus, when comparing the cell sources, PBMC-derived macrophages demonstrate higher mRNA expression of *ABCB1.* Lastly, exposure to rifampicin had no significant impact on *ABCB1* expression. However, when rifampicin-treated cells were directly compared to their untreated counterparts by the linear regression model, some rifampicin effects were observed. For instance, rifampicin enhanced *ABCB1* expression fivefold (from 0.4 ± 0.01 to 2 ± 0.9 of relative gene expression; *P* < 0.05) in M1 cells and sixfold (from 0.1 ± 0.02 to 0.6 ± 0.2 of relative gene expression; *P* < 0.05) in M2 cells, both derived from THP-1 cells. This *ABCB1* induction was confirmed at the protein level, at least for M2 macrophages (1.5 ± 0.2-fold change compared to control; *P* < 0.05). In contrast, M1 macrophages showed no significantly enhanced P-gp protein expression in rifampicin-treated cells (0.9 ± 0.2-fold change) (Fig. 3).

In PBMC-derived macrophages, neither M1 nor M2 macrophages showed significant *ABCB1* alterations after rifampicin exposure.

For *ABCG2* (Fig. 2), the three-way ANOVA showed that the cell source (PBMC vs. THP-1) is the most significant contributor (*P* < 0.0001) to *ABCG2* expression, giving the overall higher expression in PBMC-derived macrophages compared to THP-1-derived macrophages. Additionally, the cell type (M1 vs. M2) also significantly determined mRNA expression of *ABCG2* (*P* < 0.05) in untreated cells, reflected by the constantly higher *ABCG2* expression in M2 cells compared to M1 cells. Again, exposure to rifampicin showed no significant effect on drug transporter expression when data was analyzed by the three-way ANOVA. To again investigate the possible effects of rifampicin exposure selectively, treated and untreated cells within the same cell type and source were compared using the linear regression model. Only THP-1-derived M2 macrophages showed a significant two-fold enhancement of *ABCG2* mRNA expression upon rifampicin exposure (from 16.9 ± 1.2 to 34.9 ± 0.9 of relative gene expression; *P* < 0.0001).

Finally, *SLCO2B1* expression differed significantly by the cell source (P < 0.001), resulting in higher mRNA expression in PBMC-derived macrophages than in THP-1-derived macrophages. Furthermore, the cell type influenced *SLCO2B1* expression significantly (*P* < 0.05). Hence, M2 macrophages showed higher mRNA expression than M1 in the THP-1-derived macrophage subset. However, in the PBMC-derived macrophage group, this was vice versa: M1 macrophages had higher *SLCO2B1* expression levels. Once again, rifampicin exposure was not a significant contributor, neither in the three-way ANOVA analysis nor in the performed linear regression model.

A full summary of the performed three-way ANOVA and Šídák’s multiple comparisons test with the resulting *P* values can be found in Table S1, Table S2, and Table S3. Additionally, the *P* values of the performed linear regression model are described in Table S4.

## Discussion

In monocytes, rifampicin has been shown to affect *ABCB1/*P-gp expression (Asghar et al. 2002; Owen et al. 2006). In contrast, there is no such data for macrophages. In consequence, this work for the very first time evaluated the impact of rifampicin exposure on the expression levels of *ABCB1* in polarized macrophages, being derived from THP-1 cells or from PBMC. In addition, expression levels of *ABCG2* and *SLCO2B1* were assessed as well because these genes had been identified as being highly altered during macrophage differentiation and polarization (Hamburg et al. 2026). However, these two genes were hardly changed by rifampicin. Moreover, from an immunological perspective, P-gp and its expressional alteration are more relevant compared to *ABCB2* (BCRP) or *SLCO2B1* (OATP2B1). This is especially the case regarding tuberculosis therapy due to P-gp-mediated efflux of anti-tuberculosis antibiotics like bedaquiline (Kotwal et al. 2020) or moxifloxacin (Brillault et al. 2009). In this work, the data shows that *ABCB1* expression is highly dependent on the polarization (M1 vs. M2) and the source of macrophages (THP-1 vs. PBMC). Rifampicin had mixed effects. While no iatrogenic effects were seen in PBMC-derived macrophages, rifampicin-mediated *ABCB1* mRNA (in M1 and M2) and P-gp protein (M2 only) inductions were observed in THP-1-derived macrophages. The transcriptional effect sizes were quite remarkable and comparable to the alterations recorded in enterocytes or hepatocytes. For instance, exposure of LS180 cells (a colon carcinoma-derived cell line and gold standard model for *ABCB1* induction) to 10 µM rifampicin for four to 6 days enhanced *ABCB1* by 5–sevenfold (Nilles et al. 2023). In primary human hepatocytes, the same exposure doubled *ABCB1* expression (Nilles et al. 2024). This comparison not only underlines the extent of *ABCB1* induction in our THP1-derived macrophages, but also suggests that cell lines (LS180, THP-1-derived macrophages) are generally more rifampicin responsive than primary cells (hepatocytes, PBMC-derived macrophages). However, the real in vivo rifampicin effects on *ABCB1* in polarized macrophages still are hard to estimate. The PBMC-derived macrophages in fact did not show any changes of *ABCB1* expression upon rifampicin treatment, but the hypothesis cannot be conclusively rejected because the data was only taken from PBMC having been obtained from a single healthy male volunteer. In other human beings, rifampicin effects might be stronger or likewise weak. Such high interindividual variability has previously been observed in clinical investigations. For instance, when 50 healthy volunteers were treated for 1 week with daily doses of 600 mg rifampicin, the relative *ABCB1* expression in their PBMC (macrophages not investigated) varied between a 120% increase and a 5.2% decrease (compared to the pre-treatment phase) (Asghar et al. 2002). But what shall we expect if rifampicin genuinely enhanced *ABCB1*/P-gp in macrophages? Given the documented relevance of P-gp for rifampicin uptake and accumulation into macrophages (Hamburg et al. 2026), long-term rifampicin exposure could auto-induce its own efflux from macrophages, potentially diminishing its efficacy against intracellular *Mycobacterium tuberculosis*. On the flip side, this iatrogenic enhancement of P-gp activity should be reversible by P-gp inhibitors. Indeed, the calcium channel blocker verapamil has been conceptualized as an add-on during anti-tuberculosis drug therapy (Srikrishna et al. 2015; Parida et al. 2024), given its well-known P-gp-inhibitory action that translated to improved anti-tuberculosis drug efficacy, at least in mice (Gupta et al. 2013, 2015; Xu et al. 2017). More recently, we have demonstrated that rifabutin is a very potent P-gp inhibitor as well (Phondeth et al. 2024; Nilles et al. 2023; Theile et al. 2023). In consequence, adding rifabutin to rifampicin could likewise enhance rifampicin’s concentrations in plasma, tissue, or target cells such as macrophages. However, this needs further validation in pre-clinical investigations.

In conclusion, the expression of *ABCB1*, *ABCG2*, and *SLCO2B1* in macrophages strongly depends on the cell model (cell line vs. primary PBMC) and the polarization phenotype (M1 vs. M2). Moreover, rifampicin can considerably enhance *ABCB1* expression, at least in macrophages that were derived from the THP-1 monocytic cell line.

## Supplementary Information

Below is the link to the electronic supplementary material.

Supplementary file1 (DOCX 21 KB)

## Data Availability

All data supporting the findings of this study are available within the paper and its Supplementary Information.

## Abbreviations

P-gp: P-glycoprotein
PBMC: Peripheral blood mononuclear cells
