# Supplementary material for: Impact of rifampicin on P-glycoprotein (ABCB1) expression in M1 and M2 macrophages derived from the THP-1 monocytic cell line or peripheral blood mononuclear cells
Source: Naunyn Schmiedebergs Arch Pharmacol. 2026 Mar 25;399(9):14561–9. doi: 10.1007/s00210-026-05231-x (PMC13357627; doi:10.1007/s00210-026-05231-x)
Supplement: Supplementary file 1 — Supplementary file1 (DOCX 21 KB) [file 210_2026_5231_MOESM1_ESM.docx]

Table S1: Summary of performed three-way ANOVA and Šídák's multiple comparisons test for changes in mRNA expression of *ABCB1* in THP-1 and PBMC-derived M1 and M2 macrophages. Significant P values are indicated in bold.

| ***ABCB1*: Three-way ANOVA** | |
| --- | --- |
| **Source of Variation** | **P Value** |
| Cell type | **0.0003** |
| Cell source | **0.0211** |
| Treatment | 0.1604 |
| Cell type x Cell source | 0.0178 |
| Cell type x Treatment | 0.3763 |
| Cell source x Treatment | 0.3490 |
| Cell type x Cell source x Treatment | 0.7252 |
| **Šídák's multiple comparisons test** | |
| **Comparison** | **Adjusted P Value** |
| M1: THP-1-dervied Untreated vs. M2: THP-1-derived Untreated | >0.9999 |
| M1: THP-1-derived Treated vs. M2: THP-1-derived Treated | 0.7883 |
| M1: PBMC-derived Untreated vs. M2: PBMC-derived Untreated | **0.0461** |
| M1: PBMC-derived Treated vs. M2: PBMC-derived Treated | **0.0146** |
| M1: THP-1-dervied Untreated vs. M1: THP-1-derived Treated | 0.6443 |
| M1: PBMC-derived Untreated vs. M1: PBMC-derived Treated | >0.9999 |
| M2: THP-1 derived Untreated vs. M2: THP-1-derived Treated | >0.9999 |
| M2: PBMC derived Untreated vs. M2: PBMC-derived Treated | >0.9999 |
| M1: THP-1 derived Untreated vs. M1: PBMC-derived Untreated | 0.0574 |
| M1: THP-1 derived Treated vs. M1: PBMC-derived Treated | 0.5841 |
| M2: THP-1 derived Untreated vs. M2: PBMC-derived Untreated | >0.9999 |
| M2: THP-1-derived Treated vs. M2: PBMC-derived Treated | >0.9999 |

Table S2: Summary of performed three-way ANOVA and Šídák's multiple comparisons test for changes in mRNA expression of *ABCG2* in THP-1 and PBMC-derived M1 and M2 macrophages. Significant P values are indicated in bold.

| ***ABCG2*: Three-way ANOVA** | |
| --- | --- |
| **Source of Variation** | **P Value** |
| Cell type | **0.0095** |
| Cell source | **<0.0001** |
| Treatment | 0.7437 |
| Cell type x Cell source | **0.0466** |
| Cell type x Treatment | 0.5043 |
| Cell source x Treatment | 0.4479 |
| Cell type x Cell source x Treatment | 0.8085 |
| **Šídák's multiple comparisons test** | |
| **Comparison** | **Adjusted P Value** |
| M1: THP-1-derived Untreated vs. M2: THP-1-derived Untreated | >0.9999 |
| M1: THP-1-derived Treated vs. M2: THP-1-derived Treated | >0.9999 |
| M1: PBMC-derived Untreated vs. M2: PBMC-derived Untreated | 0.4812 |
| M1: PBMC-derived Treated vs. M2: PBMC-derived Treated | 0.0939 |
| M1: THP-1-derived Untreated vs. M1: THP-1-derived Treated | >0.9999 |
| M1: PBMC-derived Untreated vs. M1: PBMC-derived Treated | 0.9907 |
| M2: THP-1-derived Untreated vs. M2: THP-1-derived Treated | >0.9999 |
| M2: PBMC-derived Untreated vs. M2: PBMC-derived Treated | >0.9999 |
| M1: THP-1-derived Untreated vs. M1: PBMC-derived Untreated | 0.5145 |
| M1: THP-1-derived Treated vs. M1: PBMC-derived Treated | 0.9912 |
| M2: THP-1-derived Untreated vs. M2: PBMC-derived Untreated | **0.0137** |
| M2: THP-1-derived Treated vs. M2: PBMC-derived Treated | **0.0416** |

Table S3: Summary of performed three-way ANOVA and Šídák's multiple comparisons test for changes in mRNA expression of *SLCO2B1* in THP-1 and PBMC-derived M1 and M2 macrophages. Significant P values are indicated in bold.

| ***SLCO2B1***: **Three-way ANOVA** | |
| --- | --- |
| **Source of Variation** | **P Value** |
| Cell type | **0.0281** |
| Cell source | **0.0004** |
| Treatment | 0.2138 |
| Cell type x Cell source | **0.0096** |
| Cell type x Treatment | 0.1745 |
| Cell source x Treatment | 0.2198 |
| Cell type x Cell source x Treatment | 0.1527 |
| **Šídák's multiple comparisons test** | |
| **Comparison** | **Adjusted P Value** |
| M1: THP-1-derived Untreated vs. M2: THP-1-derived Untreated | >0.9999 |
| M1: THP-1-derived Treated vs. M2: THP-1-derived Treated | >0.9999 |
| M1: PBMC-derived Untreated vs. M2: PBMC-derived Untreated | **0.0092** |
| M1: PBMC-derived Treated vs. M2: PBMC-derived Treated | 0.9634 |
| M1: THP-1-derived Untreated vs. M1: THP-1-derived Treated | >0.9999 |
| M1: PBMC-derived Untreated vs. M1: PBMC-derived Treated | 0.1589 |
| M2: THP-1-derived Untreated vs. M2: THP-1-derived Treated | >0.9999 |
| M2: PBMC-derived Untreated vs. M2: PBMC-derived Treated | >0.9999 |
| M1: THP-1-derived Untreated vs. M1: PBMC-derived Untreated | **0.0014** |
| M1: THP-1-derived Treated vs. M1: PBMC-derived Treated | 0.3522 |
| M2: THP-1-derived Untreated vs. M2: PBMC-derived Untreated | >0.9999 |
| M2: THP-1-derived Treated vs. M2: PBMC-derived Treated | 0.9980 |

Table S4: Summary of P values of performed linear regression model depending on rifampicin treatment in THP-1 and PBMC-derived M1 and M2 macrophages. Significant P values are indicated in bold.

| **Transporter** | **Cell-type** | **Cell source** | **P Value** |
| --- | --- | --- | --- |
| ABCB1 | M1 | THP-1 | **0.041504** |
| ABCB1 | M1 | PBMC | 0.792935 |
| ABCB1 | M2 | THP-1 | **0.017592** |
| ABCB1 | M2 | PBMC | 0.891031 |
| ABCG2 | M1 | THP-1 | 0.920644 |
| ABCG2 | M1 | PBMC | 0.586340 |
| ABCG2 | M2 | THP-1 | **0.000027** |
| ABCG2 | M2 | PBMC | 0.932720 |
| SLCO2B1 | M1 | THP-1 | 0.637046 |
| SLCO2B1 | M1 | PBMC | 0.234472 |
| SLCO2B1 | M2 | THP-1 | 0.667747 |
| SLCO2B1 | M2 | PBMC | 0.648707 |
